# New Insights on Moojase, a Thrombin-Like Serine Protease from *Bothrops moojeni* Snake Venom

**DOI:** 10.3390/toxins10120500

**Published:** 2018-11-28

**Authors:** Fernanda G. Amorim, Danilo L. Menaldo, Sante E. I. Carone, Thiago A. Silva, Marco A. Sartim, Edwin De Pauw, Loic Quinton, Suely V. Sampaio

**Affiliations:** 1 Laboratório de Toxinologia, Departamento de Análises Clínicas, Toxicológicas e Bromatológicas, Faculdade de Ciências Farmacêuticas de Ribeirão Preto, Universidade de São Paulo, Ribeirão Preto 14040-903, Brazil; menaldo@fcfrp.usp.br (D.L.M.); sante.carone@gmail.com (S.E.I.C.); tasribeirao@gmail.com (T.A.S.); marcosartim@hotmail.com (M.A.S.); 2 Laboratory of Mass Spectrometry, Department of Chemistry, University of Liège, 4000 Liège, Belgium; e.depauw@ulg.ac.be (E.D.P.); loic.quinton@ulg.ac.be (L.Q.)

**Keywords:** snake venom, *Bothrops moojeni*, serine protease, thrombin-like enzyme, coagulation, fibrinogen

## Abstract

Snake venom serine proteases (SVSPs) are enzymes that are capable of interfering in various parts of the blood coagulation cascade, which makes them interesting candidates for the development of new therapeutic drugs. Herein, we isolated and characterized Moojase, a potent coagulant enzyme from *Bothrops moojeni* snake venom. The toxin was isolated from the crude venom using a two-step chromatographic procedure. Moojase is a glycoprotein with N-linked glycans, molecular mass of 30.3 kDa and acidic character (pI 5.80–6.88). Sequencing of Moojase indicated that it is an isoform of Batroxobin. Moojase was able to clot platelet-poor plasma and fibrinogen solutions in a dose-dependent manner, indicating thrombin-like properties. Moojase also rapidly induced the proteolysis of the Aα chains of human fibrinogen, followed by the degradation of the Bβ chains after extended periods of incubation, and these effects were inhibited by PMSF, SDS and DTT, but not by benzamidine or EDTA. RP-HPLC analysis of its fibrinogenolysis confirmed the main generation of fibrinopeptide A. Moojase also induced the fibrinolysis of fibrin clots formed in vitro, and the aggregation of washed platelets, as well as significant amidolytic activity on substrates for thrombin, plasma kallikrein, factor Xia, and factor XIIa. Furthermore, thermofluor analyses and the esterase activity of Moojase demonstrated its very high stability at different pH buffers and temperatures. Thus, studies such as this for Moojase should increase knowledge on SVSPs, allowing their bioprospection as valuable prototypes in the development of new drugs, or as biotechnological tools.

## 1. Introduction

*Bothrops moojeni* snake venom is a complex mixture of components, and -omics approaches have shown it to possess a great variety of different classes of toxins [1]. Venoms from Viperidae family species have large amounts of toxins with proteolytic activity, which are capable of acting on many metabolic processes, including hemostasis [2]. Among these, there are snake venom serine proteases (SVSPs), which, according to transcriptome studies, represent more than 25% of the venom transcripts in the *B. moojeni* venom gland [1]. SVSPs are usually single-chain glycoproteins with a molecular mass of between 26 and 67 kDa. This class of proteases presents a highly conserved catalytic region (His^57^, Asp^102^, and Ser^195^) and a large diversity of substrates, which provide an ability of interfering in various parts of the blood coagulation cascade, producing several hemostatic disorders [2,3,4].

SVSPs have been shown to have a functional heterogeneity in vivo. These toxins can affect several different pathways of the blood coagulation cascade, interfering with platelet aggregation, coagulation, blood pressure, complement system, and blood fibrinogen levels [5,6,7]. These various actions led to the categorization of SVSPs into different functional subtypes: thrombin-like enzymes [8], kallikrein-like [9], plasminogen activators [10], platelet aggregation inhibitors [11], protein-C activators [12], complement convertases [13], Factor-V activators [14], specific serpin inactivators [15], and prothrombin activators [16]. Although several SVSPs have been described in the literature and in spite of their high versatility, the pharmacological targets and in vivo effects of most of them are still unclear [17].

Venoms are an immense natural library of largely unexplored bioactive molecules that may contain several promising candidates for a broad range of applications [18]. Due to the different functions of SVSPs, these toxins have a great biotechnological potential and are interesting candidates for the development of new therapeutic drugs, especially for blood coagulation disorders [19]. In the literature, there are some examples of the therapeutic potential of SVSPs from *Bothrops* genus, such as Haemocoagulase^®^ from *Bothrops atrox*, commercialized by Pentapharm, which is composed of two enzymes, one with thrombin-like and the other with thromboplastin-like activities, and is indicated for the prevention and treatment of hemorrhage [20]. Regarding *B. moojeni* venom, an example is the thrombin-like enzyme Defibrase^®^ (Batroxobin), from Pentapharm [21], which has been used to treat acute cerebral infarction, unspecific angina pectoris, and sudden deafness. Concerning *Bothrops* serine proteases applied as biotechnological tools, there is Pefakit^®^ Reptilase^®^ Time, a serine protease that is useful for the valuation of fibrinogen degradation products and hypofibrinogenemias) [22,23].

Therefore, considering the rich composition of *Bothrops* venoms and the potential applications of SVSPs, this study aimed to functionally and structurally characterize a serine protease that is isolated from *B. moojeni* snake venom, named Moojase.

## 2. Results

### 2.1. Purification of Moojase

**Figure 1:**
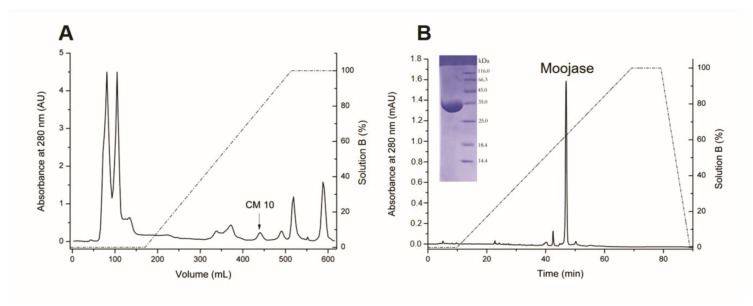
Isolation of Moojase from *Bothrops moojeni* snake venom. (**A**) Chromatographic profile of *B. moojeni* crude venom (200 mg) on a CM-sepharose column, equilibrated and eluted with 50 mM ammonium bicarbonate buffer, pH 7.8, followed by a linear gradient of up to 500 mM (solution B). Fractions were collected at a flow rate of 20 mL/h and at room temperature. (**B**) Chromatographic profile of Moojase (2.2 mg) on a C18 reversed-phase column equilibrated and eluted with 0.1% trifluoroacetic acid (TFA), and 70% acetonitrile and 0.1% TFA (solution B). Protein elution was achieved at flow rate of 1 mL/min with a linear concentration gradient of solution B. Insert, 12% SDS-PAGE of the purified Moojase under denaturing and reducing conditions.

Moojase was isolated from *B. moojeni* crude venom using a two-step chromatographic procedure. The first step was performed on a CM Sepharose cation-exchange column (Figure 1A). Then, the serine protease active fraction CM10 was submitted to the next chromatographic step with C18 reversed-phase chromatography (Figure 1B), resulting in a major fraction identified as the active serine protease Moojase by TAME (Na-p-tosyl-l-arginine methyl ester) activity. SDS-PAGE analysis of the Moojase, under non-reducing conditions, revealed a single band (Figure 1B inset).

The purification process for Moojase yielded 1.8 mg of the purified protein after the reversed phase chromatography, which represents a 0.9% recovery from the 200 mg of crude venom. Moojase showed a specific activity of 11,226.0 U/mg over the TAME substrate, representing a 5.08-fold purification (Table 1). Also, to confirm the serine protease activity, we incubated Moojase with PMSF, which resulted in the inhibition of its TAME activity (data not shown).

### 2.2. Isoelectric Focusing

Isoelectric focusing of Moojase resulted in four protein bands, indicating different pI values for this serine protease, ranging from 5.80 to 6.88. The major band showed a pI of 6.49 (Figure S1).

### 2.3. Structural Characterization

The theoretical molecular mass of Moojase, as predicted by Sequence Editor software for its oxidized form, was 25.4 kDa, while the analysis by Fourier Transform Ion Cyclotron resonance mass spectrometer revealed a practical molecular mass of 30.3 kDa (Figure S2). This difference of 4.6 kDa between the practical and theoretical masses may be due to glycosylation, which is not considered in the current theoretical prediction.

**Figure 2:**
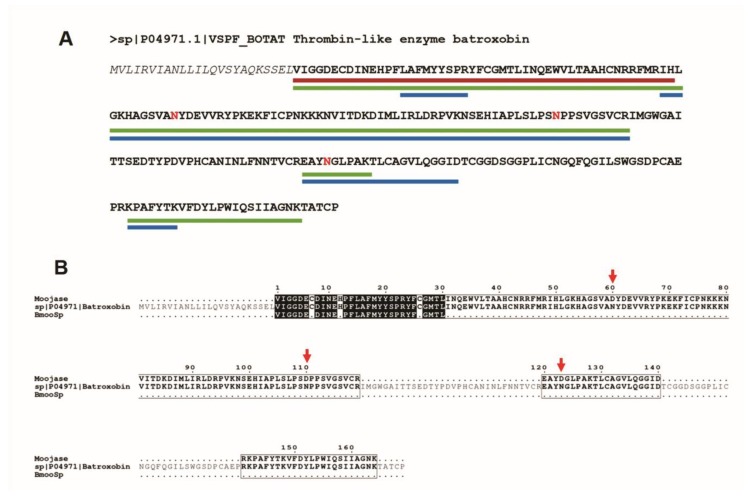
Moojase sequencing by three different techniques. (**A**) Batroxobin (P04971.1) was used as a template, and the fragments identified are marked with coloured lines: red line for Edman degradation sequencing, green line for MALDI-TOF and blue line for Q-Exactive mass spectrometry. The amino acids in red are those that the software Peaks Studio identified by the punctual mutation on de novo sequencing (SPIDER algorithm). (**B**) Alignment of the Moojase fragments identified in our study against the sequences of Batroxobin (P04971.1) and BmooSP [4]. The red arrow indicates the punctual mutations in the Moojase sequence. The black boxes represent the conserved amino acids in all sequences. Black residues are conserved in at least two sequences, while non-conserved residues are represented in gray. The black dots represent the gaps in the sequence which were not sequenced.

To perform the sequencing of Moojase, three different methodologies were applied, which resulted in the first 50 amino acids from the N-terminus by Edman degradation, 151 residues by MALDI-TOF (Matrix Assisted Laser Desorption/Ionization-Time of Flight), and 188 amino acid sequenced by Q-Exactive mass spectrometry techniques. Considering the Batroxobin sequence as a template, the determined amino acid residues represent 21.4% (Edman degradation), 64.5% (MALDI-TOF) and 80.3% (Q-Exactive) of its full amino acid sequence (with 234 residues). The identified fragments are summarized in Figure 2, and the data from mass spectrometry analysis are detailed in the Figure S3.

Looking for punctual mutations, *de novo* sequencing was performed by the SPIDER algorithm from Peaks Studio software. The results are summarized using the template sequence (Batroxobin—P04971.1) in Figure S3. These mutations are highlighted in red in Figure 2A, consisting of asparagine (N) residues that were mutated to aspartate (D). These differences in the sequence and those fragments that were not sequenced in Batroxobin may be evidence that Moojase represents an isoform of Batroxobin, as we can observe in the alignment presented in Figure 2B.

### 2.4. Glycosylation Analyses

**Figure 3:**
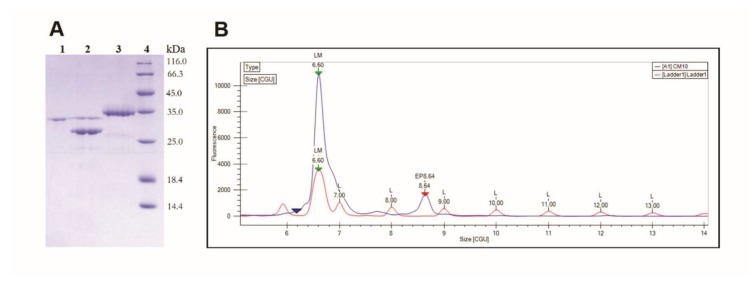
Glycosylation analyses. (**A**) Deglycosylation of Moojase (20 µg) was performed using a PNGase F deglycosylation kit, followed by evaluation on 12% SDS-PAGE under reducing conditions. Lanes: 1. PNGase F, 2. PNGase F + Moojase; 3. Moojase; 4. Molecular mass standard. (**B**) Electropherogram of the glycan portion of Moojase by capillary electrophoresis. The electropherogram is presented as CGU (caliper glucose units) versus fluorescence intensity. CGU values are obtained from the standard profile (Ladder1, shown in red), which consists of a standard amount of glucose oligomers. Then, standard (red) and sample (blue) separations are aligned through the addition of a lower marker (6.60 CGU peak), which overlaps the electropherograms and relates them. Moojase glycan appeared as a single major peak with 8.64 CGU.

Deglycosylation of Moojase with PNGase F resulted in the reduction of its molecular mass from 36.6 kDa to 30.3 kDa, a difference of 6.3 kDa that should correspond to N-linked glycans (Figure 3A). In addition, in the LabChip electropherogram of Moojase, only a single major peak could be observed, with 8.64 CGU (caliper glucose units) (Figure 3B). This allows us to suggest that the glycan portion of Moojase is composed of one polysaccharide species with a relative mass of 8.64 glucose monomers.

### 2.5. Coagulant Effects

**Figure 4:**
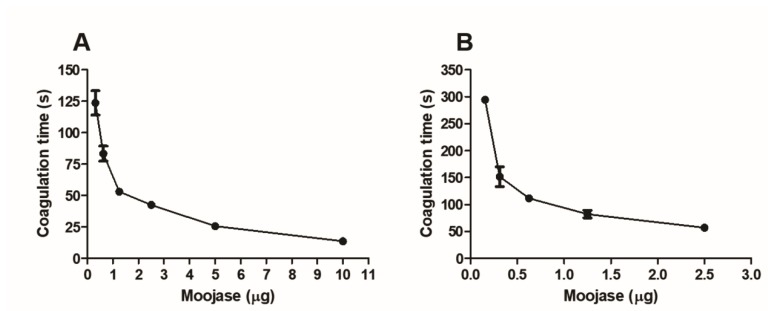
Coagulant activity of Moojase. (**A**) Coagulation of human plasma. Human-citrated platelet-poor plasma (200 µL) was incubated at 37 °C in the presence of different doses of Moojase, observing the reactions to determine the time (in seconds) required to the clot formation. (**B**) Coagulation of fibrinogen solutions. Different doses of Moojase were incubated with 200 µL of human fibrinogen solution (3 mg/mL) at 37 °C, followed by the observation of the time (in seconds) required to the clot formation. Results expressed as means ± SEM of two independent experiments (*n* = 3).

Moojase was able to clot platelet-poor plasma (Figure 4A) and fibrinogen solutions (Figure 4B) in a dose-dependent manner, which indicates thrombin-like properties.

### 2.6. Fibrinogenolytic Effects

**Figure 5:**
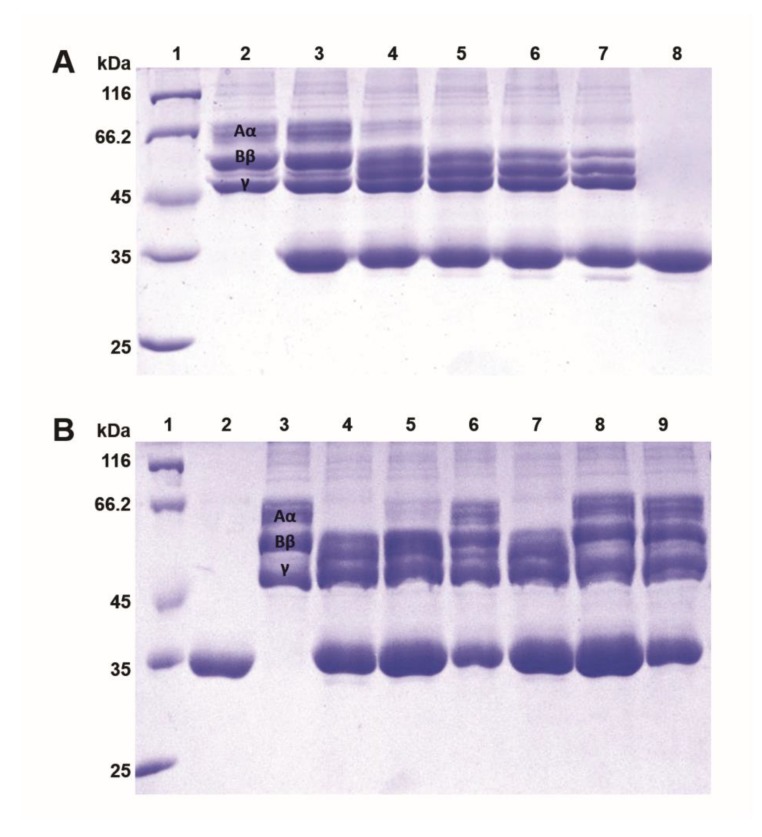
Fibrinogenolytic activity of Moojase. Analysis was made on 12% SDS-PAGE after incubation of Moojase (5 µg) with human fibrinogen (15 µg) at 37 °C for different time periods or after preincubation with different inhibitors (20 mM) for 30 min. (**A**) Lanes: 1. Molecular mass standard; 2. Fibrinogen control; 3. Fibrinogen + BjSP (0 min); 4. Fibrinogen + Moojase (10 min); 5. Fibrinogen + Moojase (30 min); 6. Fibrinogen + Moojase (60 min); 7. Fibrinogen + Moojase (120 min); 8. Moojase. (**B**) Lanes: 1. Molecular mass standard; 2. Moojase; 3. Fibrinogen control; 4. Fibrinogen + Moojase (120 min); 5. Fibrinogen + Moojase + Benzamidine (120 min); 6. Fibrinogen + Moojase + PMSF (120 min); 7. Fibrinogen + Moojase + EDTA (120 min); 8. Fibrinogen + Moojase + SDS (120 min); 9. Fibrinogen + Moojase + DTT (120 min).

Moojase rapidly induced the proteolysis of the Aα chains of human fibrinogen, followed by the degradation of the Bβ chains after longer incubation periods (Figure 5A). When the enzyme was preincubated with different inhibitors (Figure 5B), we can see that PMSF (an irreversible serine protease inhibitor), SDS (a denaturing agent), and DTT (a reducing agent) were all able to prevent the degradation of both chains of fibrinogen, while benzamidine (a reversible serine protease inhibitor) and EDTA (a chelating agent) did not inhibit the fibrinogenolytic effects of Moojase.

**Figure 6:**
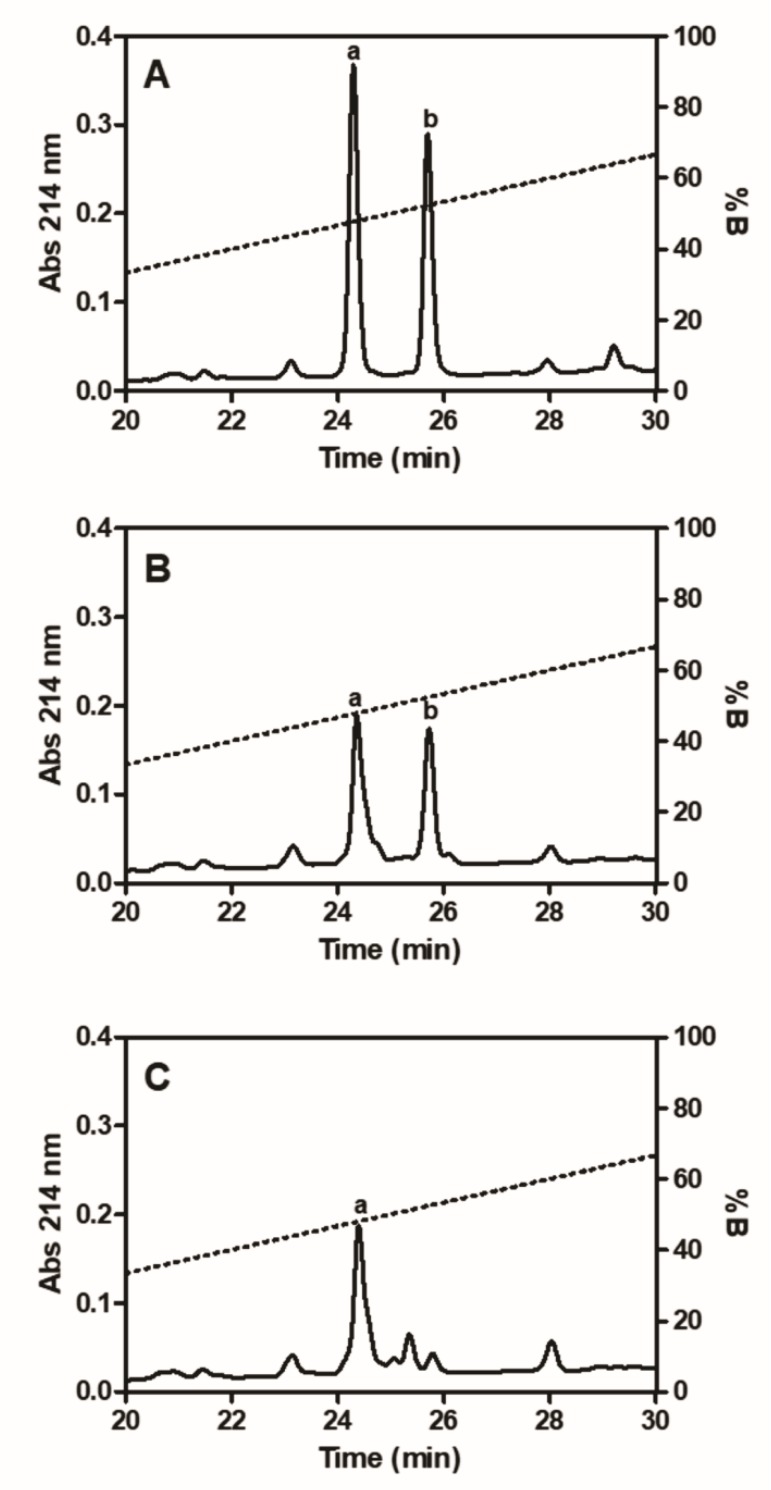
RP-HPLC analysis of fibrinopeptides. Standard fibrinopeptides A (*a*) and B (*b*), used for comparative purposes (**A**) and fibrinopeptides formed by incubating human fibrinogen (3 mg/mL) with thrombin (5 µg/mL); (**B**) or Moojase (20 µg/mL); (**C**) at 37 °C for 120 min were analyzed by reversed phase HPLC at 214 nm.

Besides the fibrinogenolysis evaluation by SDS-PAGE, RP-HPLC analyses were also used to determine the formation of fibrinopeptides by Moojase (Figure 6). Using the purified fibrinopeptides (Figure 6A) and those formed by thrombin (Figure 6B) as controls and at the conditions assayed (Moojase at 20 µg/mL with fibrinogen at 3 mg/mL for 2 h at 37 °C), our results showed that the serine protease mainly generated fibrinopeptide A (Figure 6C).

### 2.7. Fibrinolytic Effects

Moojase was also able to induce the fibrinolysis of fibrin clots formed in vitro (Table 2). When compared to plasmin (used as a positive control of fibrinolysis), Moojase at the same dose (20 µg) induced a larger fibrinolytic halo, indicating a more potent fibrinolytic activity at the assayed conditions.

### 2.8. Induction of Platelet Aggregation

Moojase at 20 µg induced around 71% aggregation of washed platelets (Table 3), which is a higher effect when compared to that of ADP (a platelet aggregation agonist) at the same dose.

### 2.9. Effects on Chromogenic Substrates

**Figure 7:**
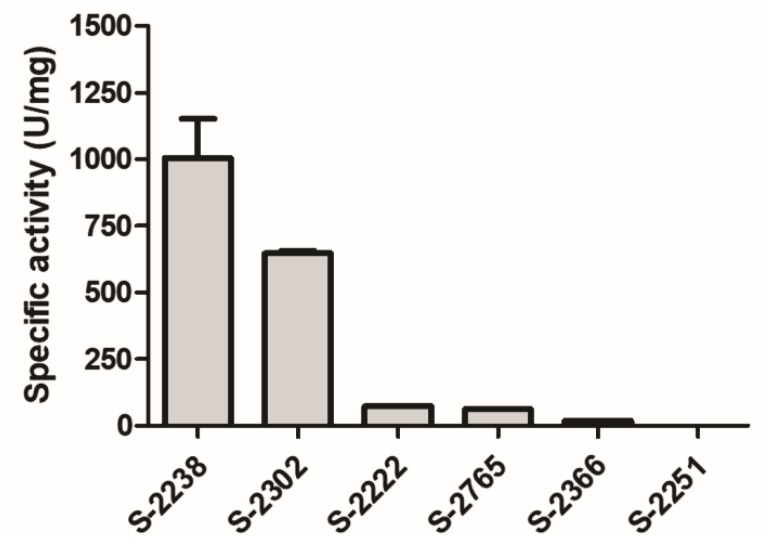
Effects of Moojase on different chromogenic substrates. Moojase (2 µg) was incubated for 40 min at room temperature with different chromogenic substrates (0.4 mM, final concentration) and the reactions were monitored at 405 nm. Absorbance values were then expressed as specific activity (U/mg of Moojase). Results expressed as mean values ± SEM (*n* = 3).

Moojase showed high cleavage effectiveness for substrates S-2238 (used to determine the activity of thrombin) and S-2302 (used for plasma kallikrein, factor XIa and factor XIIa) (Figure 7). These results reinforce our previous findings, indicating that this serine protease is a thrombin-like enzyme. In addition, effects on substrate S-2302 indicate that Moojase should also act on different components of the coagulation cascade other than fibrinogen.

### 2.10. Stability Studies

**Figure 8:**
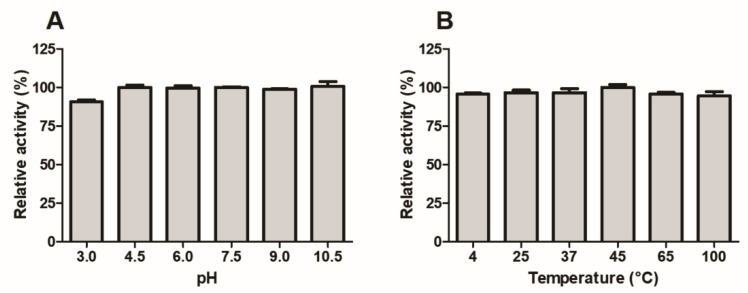
Effects of variations of pH (**A**) and temperatures (**B**) on the TAME esterase activity of Moojase. The serine protease (5 µg) was initially incubated at different pH values and temperatures for 30 min, followed by reaction with TAME (1 mM, final concentration) for 30 min at 37 °C. Absorbances were obtained at 247 nm, and the results were expressed as mean values ± SEM (*n* = 3) of relative activities (%).

Figure 8 shows that the esterase activity of Moojase was practically unaffected, even after 30 min of incubation at different pH buffers (Figure 8A) and temperatures (Figure 8B), with the only reduction being observed at pH 3.0 (Figure 8A). These results demonstrate that Moojase is a serine protease with very high stability.

**Figure 9:**
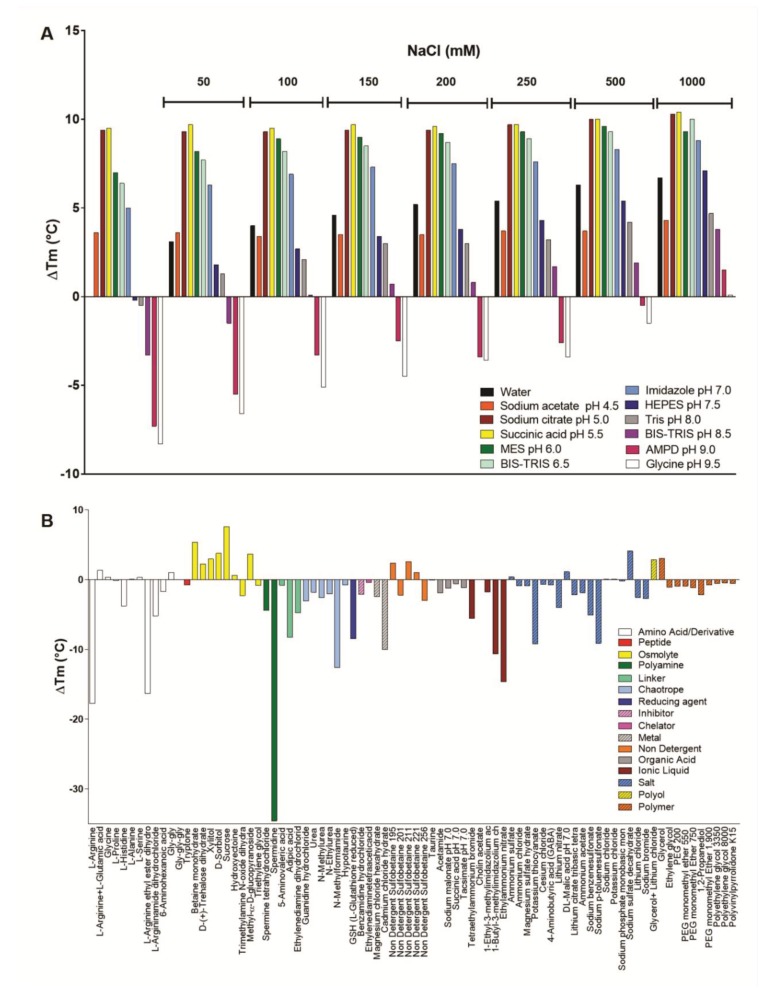
Thermostability of Moojase as analyzed by thermofluor. (**A**) pH and salt concentration screening and (**B**) thermostability assay of Moojase performed using a screening kit containing different classes of chemical additives. Results are presented as the variation of the protein melting temperature measurement (∆Tm).

In order to evaluate the effects of pH, salt concentration, and chemical additives on Moojase stability, the thermofluor technique was also employed, which provides a full screening at different conditions, and with high sensibility. Initially, pH and salt concentration screenings were performed (Figure 9A), showing that Moojase exhibited the highest thermostability at conditions with the pH varying from 5.0 to 5.5. This fact corroborates the results described in Figure 8A. In parallel, as the sodium chloride concentration increases, the thermostability of Moojase also rises at all pH conditions, maintaining the highest ∆Tm values at pH 5.5 (Figure 9A).

Considering the results obtained above, a subsequent thermostability assay with Moojase was performed using a screening kit containing different classes of chemical additives. An overall analysis showed that most chemical agents decreased Moojase thermostability (Figure 9B). In the presence of amino acids or its derivates, Moojase interestingly presented a decrease in thermostability in most conditions with a prominent effect in the presence of l-arginine (−17.74 °C) and its derivate l-arginine ethyl ester dihydrochloride (−16.35 °C), followed by l-argininamide dihydrochloride (−5.15 °C) (Figure 9B). Another highlight concerns the polyamine agent spermidine, which induced the stability decrease of −34.57 °C. The chemical agents, classes of linkers, chaotropic agents, metals, ionic liquids, and specific salts were also able to induce a notable decrease in Moojase thermostability (Figure 9B). Aside from the osmolyte class, the effect of some singular random agents is also worth mentioning. As observed in Figure 9B, benzamidine and EDTA presented a modest capacity to reduce Moojase thermostability (−2.9 and −0.4 °C, respectively). Regarding the chemicals that increased Moojase thermostability, most osmolyte agents were responsible for this effect, led by sucrose (+7.62 °C). Some random thermostabilizing results were observed for non-detergent agents (sulfobetaine 195, 211, and 221), a salt (sodium sulfate decahydrate), and a polyol (glycerol alone and in the presence of lithium chloride) (Figure 9B).

## 3. Discussion

Serine proteases are ubiquitous enzymes that are present in the venom of several snake species [17,24,25,26]. These proteases are glycoproteins, synthesized as zymogens, which present a single peptide chain and variable molecular masses, depending on their glycosylation levels. SVSPs display an extremely conserved catalytic triad (His^57^, Asp^102^ and Ser^195^), and they present the ability to act on various parts of the blood coagulation cascade [2,27]. Although most SVSPs are fibrinogen-clotting enzymes, some studies have also described SVSPs that did not promote coagulation [24,28,29].

Several studies reported the isolation and functional characterization of toxins from this protein class, including some from *B. moojeni* venom [4,30,31]. However, regarding those from this snake species, there are only two reports in which part of the amino acid sequence of the proteases were described [4,30]. Fernandes de Oliveira et al. [32], in 2013, identified two isoforms of serine proteases, named BMII32 and BMII35, with molecular masses that were close to 32 and 35 kDa, respectively. BMII32 and BMII35 were shown to present plasma clotting activity and fibrinogenolytic action without inducing fibrinolysis. A few years later, the same research group isolated a serine protease from *B. moojeni* venom, named BmooSP, with a molecular mass of 36 kDa in its reduced form, and 32 kDa in its oxidized form [4]. BmooSP showed coagulant and defibrinating activities in vitro, and caseinolytic and fibrinogenolytic actions in vivo. The sequencing of these serine proteases (BMII32, BMII35 and BmooSP) revealed high identity to Batroxobin. In our study, we described a similar serine protease and named it Moojase, which was purified in two chromatographic steps, and showed high enzymatic activity on a TAME substrate, and was inhibited by PMSF. In addition, Moojase presented a molecular mass of 30.3 kDa and was shown to be a glycoprotein with a N-glycan portion of 8.64 CGU.

Some serine proteases from South American *Bothrops* snakes were named Batroxobin, with the first being described as early as 1939 [33]. These enzymes have been widely used in the pharmaceutical industry, as defibrinogenating drugs or as reagents for the diagnosis of dysfibrinogenemias. Batroxobin from *B. atrox* snake venom is used as a hemostatic drug (Reptilase), since it mediates the formation of fibrin I, together with thrombin, which makes blood more prone to coagulate. The Batroxobin isolated from *B. moojeni* snake venom is used as a defibrinogenating agent (Defibrase). Its defibrinogenating action is associated to the formation of fibrin I from fibrinogen, and with the release of tissue plasminogen activator (t-PA) from the endothelium, which also leads to fibrinolysis [27]. Herein, we used the Batroxobin sequence as a template to aid with the sequencing of Moojase. Although it was not possible to determine the full primary sequence composition of Moojase, from our results, we may consider it as a new isoform of Batroxobin. Emphasizing this hypothesis, the isoelectric focusing analysis of Moojase revealed four different pI values: 5.80, 6.13, 6.49, and 6.88. De Oliveira et al. [4] described two different pI values for BmooSP, including those described herein (6.13 and 6.49), while Batroxobin from *Bothrops moojeni* showed a pI of 6.6. Taken together, these data suggest that all these serine proteases are very similar to each other, and may represent isoforms.

The micro-heterogeneity is common between proteins from snake venoms, as already discussed by Oliveira et al. [4]. Our results concerning the similarities among Moojase, BmooSP, and Batroxobin represent a good example of this micro-heterogeneity in the serine protease class. This micro-heterogeneity can result from variations in internal sequences, in the number or types of carbohydrate domains in glycosylation, or from other post-translational modifications of these toxins in the venom gland [4,34,35,36,37].

Disturbances in blood coagulation are among the most severe effects of envenomations by viperid snakes [27]. Our results showed that Moojase displayed significant effects on hemostasis, being able to coagulate platelet-poor plasma and fibrinogen solutions, also inducing fibrin(ogen)olysis and platelet aggregation. Moojase cleaved the Aα and Bβ chains of fibrinogen, with greater affinity to the Aα chains, as shown by the main formation of fibrinopeptide A. This action on fibrinogen should be related to the strong coagulation that it induced in platelet-poor plasma. In addition, this enzyme showed high amidolytic activity on chromogenic substrates for thrombin (S-2238) and kallikrein (S-2302), and induced the aggregation of washed platelets in the presence of calcium.

All of the above-mentioned effects of Moojase are in agreement with a thrombin-like behaviour, which is a common feature of SVSPs. Although most SVSPs are classified as thrombin-like enzymes, they often do not present all of the functions of thrombin. One of the main differences between thrombin and SVSPs is the formation of abnormal fibrin clots by the latter, due to the inability of most SVSPs to generate factor XIIIa (exceptions include Ancrod, which was shown to activate factor XIII, forming solid fibrin clots [38,39,40]), resulting in the inhibition of normal blood coagulation on victims [41,42]. These characteristics of SVSPs emphasise their therapeutic potential as defibrinogenating agents [43].

In addition to its promising biological effects, stability studies showed that Moojase is highly stable at different temperatures and pH values. In order to better understand some physical–chemical properties of Moojase, we have performed the thermofluor assay of this toxin in the presence of different conditions of pH, salt concentrations and chemical additives. This technique assesses the effect of solvent conditions and ligands on the protein thermostability, based on temperature-dependent protein unfolding [44,45]. Thermofluor assay is composed by two high-throughput screening kits in which an initial screening for global parameters is performed, followed by a search for protein-specific additives. The first screening allows to determine global stability tendencies according to pH, salt concentration, buffer type and concentration. The second assay contains small molecules that can affect the folding, aggregation state, and solubility or it can specifically bind and stabilize proteins [46].

Thermofluor assays present several applications, and they can be very useful in toxinology in order to evaluate the purification procedure and storage protocols, also helping to optimize these protocols [46,47]. Moojase showed the highest ∆Tm at pH 5.5, with increasing thermostability in the presence of sodium chloride up to 1 M. This could be associated with the salting-in phenomenon, considering that thermostability and solubility can have a positive correlation [48,49].

Among the classes of agents assessed, osmolytes presented the most significant effects on enhancing Moojase thermostabilization. Sugar and sugar-based polyol molecules are well-known osmolytes that protect against protein denaturation, working as “chemical chaperones” [50]. The mechanism by which sorbitol and sucrose act as protective osmolytes involves protein backbone side-chain amino acid interactions with osmolytes, and solvents inducing a folding conformation with a minimum possible exposed surface area [51].

Interestingly, the amino acid l-arginine and its derivates promoted a decrease in Moojase thermostability. This effect could be associated with the capacity of arginine to be a chaotropic agent that is responsible for the disruption of the hydrogen bonding between water molecules, therefore destabilizing the protein solubility [52]. Moreover, other chaotropic agents assessed in the thermofluor assay also decreased Moojase thermostability. Another important aspect observed was that spermidine induced the highest ∆Tm shift, showing a decrease in thermostability. Spermidine is a low molecular weight polyamine that can bind to proteins and alter their stability and structure [53].

## 4. Conclusions

In summary, the present study described the results of isolation, and structural and functional characterization of Moojase, a thrombin-like serine protease from *B. moojeni* snake venom. Moojase was shown to be a potent coagulant toxin, inducing significant fibrin(ogen)olysis, and thus being an interesting candidate for defibrinogenating and/or thrombolytic agents. Knowledge about SVSPs represents an advancement in the characterization of these enzymes, allowing their bioprospection as valuable prototypes in the development of new drugs or as biotechnological tools.

## 5. Material and Methods

### 5.1. Venom

Desiccated *B. moojeni* venom was provided by the Center for the Study of Venoms and Venomous Animals (CEVAP) from São Paulo State University (Botucatu, SP, Brazil).

### 5.2. Human Plasma

The human plasma used in this study was obtained from the blood of 20- to 40-year-old healthy volunteers, both male and female, after due approval by the Research Ethics Committee of the School of Pharmaceutical Sciences of Ribeirão Preto (CEP/FCFRP approval n. 694.165, CAAE: 26264714.0.0000.5403, obtained in 23 June 2014). Participating donators agreed to be part of this research, and gave a written informed consent allowing for the publication of data resulting from their blood samples. Blood was collected by venipuncture using 3.8% sodium citrate (9:1, *v/v*) as anticoagulant, centrifuged at approximately 200× *g* for 15 min at room temperature to obtain platelet-rich plasma (PRP) or at approximately 1050× *g* for 15 min at room temperature to obtain platelet-poor plasma (PPP). These plasma samples were then used in the coagulant and platelet aggregation assays described below.

### 5.3. Isolation of Moojase

Moojase was purified by two chromatographic steps. Initially, 200 mg of crude venom was applied to a Carboxymethyl (CM) Sepharose cation-exchange column (26 × 2 cm, GE Healthcare, Chicago, IL, USA)), equilibrated, and eluted with 50 mM ammonium bicarbonate buffer (NH_4_HCO_3_), pH 7.8, followed by a linear gradient up to 500 mM at a flow rate of 20 mL/h. The fraction of interest was then submitted to reverse-phase chromatography on a C18 column (CLC-ODS, 0.46 × 25 cm, Shimadzu, Kyoto, Japan) using a HPLC system (Shimadzu). The column was equilibrated with 0.1% trifluoroacetic acid (TFA, solvent A) and elution was carried out at a flow rate of 1 mL/min and a linear concentration gradient of 70% acetonitrile and 0.1% TFA (solvent B), as follows: 0% B for 0–10 min, 0–100% B for 10–70 min, and 100% B for 70–80 min. All peaks were monitored by measuring the absorbance at 280 nm. Protein concentrations were determined by the Pierce™ BCA Protein Assay Kit (Thermo Fischer Scientific, Waltham, MA, USA), following the manufacturer’s instructions.

### 5.4. Electrophoresis and Isoelectric Focusing

The purification efficiency and evaluation of deglycosylation were monitored by sodium dodecyl sulphate polyacrylamide gel electrophoresis (SDS-PAGE), using 12% gels, and staining with Coomassie blue R-250, as described by Laemmli [54].

2D electrophoresis was also performed, and consisted of an isoelectric focusing step, followed by SDS-PAGE analysis. For the first dimension, 20 µg of Moojase was prepared in 250 µL of DeStreak Rehydration Solution (GE Healthcare, 17-6003-19, Chicago, IL, USA) with 0.5% IPG Buffer, pH 3-10 (GE Healthcare, 17-6000-87, Chicago, IL, USA), and then incubated with a 13 cm Immobiline DryStrip pH 3-10 (GE Healthcare, 17-6001-14, Chicago, IL, USA) overnight. After rehydration, the strip was applied to an isoelectric focusing system (Ettan IPGphor 3, GE Healthcare, Chicago, IL, USA) and the focusing was carried at 50 µA as follows: Step 1, 500 V, 1 h, and 0.5 kVh; Step 2, gradient 500 to 1000 V, 1 h and 0.8 kVh; Step 3, gradient 1000 to 8000 V, 2.3 h and 11.3 kVh; Step 4, 8000 V, 10 to 30 min and 1.4 to 4.4 kVh. For the second dimension, the strip was washed with DTT diluted in 40 mL of equilibration buffer solution (6 M urea, 30% glycerol, 2% SDS, 50 mM Tris-HCl, pH 7.4, 0.002% bromophenol blue) for 15 min, and then washed with iodoacetamide diluted in 40 mL of the same buffer for 15 min. The strip was then applied to a 15% polyacrylamide gel, and electrophoresis was carried with max 600 V and 100 W and 15 mA for 15 min, and then 60 mA for 5 h. After that, the gel was stained with Coomassie Blue R-250, scanned, and analyzed using the Melanie 9 program (GE Healthcare, Chicago, IL, USA).

### 5.5. Serine Protease Activity

The esterase activity of the Moojase was tested using the substrate Na-p-tosyl-l-arginine methyl ester (TAME), according to the previously described methodology [55] with modifications [6]. Briefly, absorbance was monitored at 247 nm after 30 min of reaction of the samples (10 µg) with 1 mM TAME (final concentration) at 37 °C, expressing the results as specific activity, related to TAME units per milligram of protein (U/mg). In addition to the chromatographic fractions and Moojase, TAME activity of samples after incubation with PMSF (phenylmethylsulfonyl fluoride) at 37 °C for 30 min was also evaluated.

### 5.6. Structural Characterization of Moojase

#### 5.6.1. Molecular Mass Determination

The samples were solubilized in a solution of acetonitrile/water/formic acid (49.8/50/0.2 *v/v*) for molecular mass determination. The mass spectrometric analyses were executed in a Bruker Solarix 9.4T FTICR mass spectrometer (Bruker Daltonics, Bremen, Germany). Positive ions were generated for nano-ESI (electrospray ionization) with a NanoMate (Advion Biosciences, Ithaca, NY, USA). Ions were transferred into the spectrometer through a quadrupole followed by accumulation of data during 1 s in the hexapole. Afterwards, the ions entered the ICR cell, where they were submitted to dynamic trapping. The acquisition of spectra was performed in a mass range of *m*/*z* 70–2000 during a transient for which 2 M points provided a mass resolving power around 100,000 (at *m*/*z* 400), after FFT processing (the total time per scan was 2 s). CID (collision-induced dissociation) fragments of Glu-1-fibrinopeptide B was used as an external calibration. DataAnalysis 4.0 software (Bruker Daltonics, Bremen, Germany) was applied to processed and to analyse the mass spectra.

#### 5.6.2. Amino Acid Sequencing

Sequencing of the primary structure of Moojase was accomplished by three different techniques: Edman degradation, mass spectrometry through MALDI-TOF/TOF (Ultraflextreme, Bruker, Billerica, MA, USA.), and Q-Exactive Hybrid Quadrupole-Orbitrap Mass Spectrometer (Thermo Scientific, Waltham, MA, USA) for de novo sequencing. N-terminal sequencing was performed by Edman degradation [56], using an automated PPSQ-33A sequencer (Shimadzu Co., Kyoto, Japan). The search for sequential identities was carried out by using BLAST from NCBI.

Full-length sequence of amino acids was sequenced by MALDI-TOF/TOF mass spectrometry. Therefore, Moojase was first subjected to reduction, alkylation, and enzymatic digestion. The toxin was reduced with 2 µL of 0.1 M dithiothreitol (DTT) and 6 µL of 0.5 M NH_4_HCO_3_, over 1 h at 58 °C. The samples were then alkylated with 2 µL of 0.5 M iodoacetamide (IAA) and incubated at 37 °C during 1 h, in the dark. The toxin was finally digested by the porcine trypsin and Glu-C enzymes (at 37 °C over 2 h). After each process, the reaction was stopped by 1% formic acid (final concentration), subjected to desalting by C18 ZipTip columns and eluted in a solution of acetonitrile/water/formic acid (49.8/50/0.2 *v/v*). Aliquots were collected after each step and the peptide mass fingerprint (PMFs) were analyzed by MALDI-TOF (Ultraflex II, Bruker, Billerica, MA, USA) using 2,5-DHB as the matrix operated in positive reflectron mode. The most intense parent ions were selected to be fragmented by post-source decay activation, and sequenced to confirm the amino acid sequence. The mass spectra obtained were analyzed using FlexAnalysis 3.0, BioTools 3.2 and Sequence Editor bioinformatics software tools.

The digested material was also analyzed using a UPLC nanoACQUITY (Waters, Milford, MA, USA) coupled to a Q-Exactive Hybrid Quadrupole-Orbitrap Mass Spectrometer (Thermo Scientific, Waltham, MA, USA). The chromatographic system is equipped with a monolithic capillary column (PepSwift, 100 µm × 5 cm, Thermo Scientific, Waltham, MA, USA). Elution of peptides was performed with a gradient of 3–50% solution B for 80 min (A: water/0.1% formic acid; B: acetonitrile) at a flow rate of 0.7 mL/min, and the acquisitions were carried out in positive mode. De novo sequencing of Moojase was performed with PEAKS Studio 8.5 software [57], with “Serine Protease” from the UniProt database, downloaded in June 2018. Therefore, in the software, some parameters were selected: carbamidomethylation as fixed modification and amidation/oxidation (M) (methionine oxidation) as variable modifications. We considered the maximum missed cleavages of 3, and the parent mass and fragment mass error tolerance were set at 5 ppm and 0.015 Da, respectively. Unique peptide ≥2 and false discovery rate (FDR) of 1% were used for filtering out inaccurate proteins for the SPIDER search algorithm. Only peptides with −10logP > 20 were used to detect the proteins from the database.

### 5.7. Deglycosylation Analysis

Moojase (20 µg) was subject to deglycosylation analysis using the PNGase F deglycosylation kit (Promega, Madison, WI, USA), following the protocol for “Protein Deglycosylation Using Non-Denaturing Conditions” and the manufacturer’s guidelines. After the deglycosylation, Moojase was submitted to 12% SDS-PAGE.

The glycan portion of Moojase was also analyzed using the LabChip GXII Touch (PerkinElmer, Waltham, MA, USA) equipment. Protein samples were submitted to preparation according to the ProfilerPro Glycan kit, which uses PNGase F to deglycosylate the samples, followed by fluorescent labeling of the free N-linked carbohydrates. Labeled glycans were then separated by microchip capillary electrophoresis and an electropherogram was plotted with the resulting signals, together with a standard composed of a mixture of glucose oligomers with different numbers of glucose molecules. Analysis of the results was made in the LabChip GX Reviewer analysis program.

### 5.8. Coagulant Activity

#### 5.8.1. Clotting of Human Plasma

Human-citrated platelet-poor plasma (200 µL) was incubated at 37 °C in the presence of 10 µL of solutions containing 0.3125, 0.625, 1.25, 2.5, 5.0, or 10.0 µg of Moojase. Reactions were observed visually to determine the time (in seconds) required to the clot formation.

#### 5.8.2. Clotting of Fibrinogen Solutions

Fibrinogen clotting was assayed as previously described [58], with minor modifications. Briefly, solutions of 10 µL containing 0.156, 0.3125, 0.625, 1.25, or 2.5 µg of Moojase were incubated with 200 µL of human fibrinogen solution (3 mg/mL) at 37 °C to observe the time (in seconds) required for clot formation.

### 5.9. Fibrinogenolytic Activity

The fibrinogenolytic activity of Moojase was determined as previously described [59], with modifications. Initially, the serine protease (5 µg) was incubated with human fibrinogen (15 µg in 50 mM Tris-HCl buffer, pH 7.4, containing 70 mM NaCl) at 37 °C for 0, 10, 30, 60, and 120 min. Then, the enzyme was also preincubated with the inhibitors benzamidine (20 mM) and ethylenediaminetetraacetic acid (EDTA, 20 mM), phenylmethylsulfonyl fluoride (PMSF, 20 mM), dodecyl sodium sulfate (SDS, 20 mM), and dithiothreitol (DTT, 20 mM) for 30 min at 37 °C prior to the addition of fibrinogen and incubation at 37 °C for another 120 min. After this, the denaturing buffer containing β-mercaptoethanol was added to the samples, followed by heating at 100 °C for 5 min. Then, samples were subjected to 12% SDS-PAGE for the fibrinogen degradation analysis.

### 5.10. Identification of Fibrinopeptides

Fibrinopeptides generated by the proteolytic activity of Moojase were identified, as previously described [24]. Briefly, human fibrinogen (3 mg/mL in 50 mM Tris-HCl buffer, pH 7.4, containing 70 mM NaCl) was incubated at 37 °C for 120 min with Moojase (20 µg/mL). Fibrinopeptides were analyzed by reversed-phase high performance liquid chromatography (RP-HPLC) at 214 nm, using a C18 column (0.46 × 25 cm, CLC-ODS, Shimadzu, Japan) and a gradient of solutions A (0.1% TFA) and B (70% acetonitrile in 0.1% TFA). Purified fibrinopeptides A and B (Sigma F3254 and F3379) were chromatographed to determine their elution times and to use them as a reference. Fibrinopeptides formed by the incubation of thrombin (5 µg/mL) with fibrinogen for 120 min were evaluated as a positive assay control.

### 5.11. Fibrinolytic Activity

The fibrinolytic activity of Moojase was assessed on fibrin clots formed in Petri dishes, according to the methodology of Leitão et al. [60]. Samples of Moojase (5 and 20 µg), phosphate-buffered saline (PBS, negative control), and plasmin (20 µg, positive control) were applied to the fibrin gel cavities, and the plate was incubated for 24 h at 37 °C. After that period, the fibrinolysis halos were measured and expressed in millimeters (mm).

### 5.12. Platelet Aggregation Assays

Preparation of washed platelets essentially followed the methodology described by Lopes-Pires et al. [61]. Platelet aggregation was evaluated by turbidimetry using a platelet aggregometer (Chrono-log Corporation, model 490 2D, Havertown, PA, USA) and the software AggroLink. A mixture containing washed platelets (440 µL) and Moojase (20 µg) was incubated for 10 min at 37 °C, and its turbidity was monitored in an aggregometer. ADP (adenosine diphosphate) was used as a platelet aggregation agonist.

### 5.13. Amidolytic Activity on Chromogenic Substrates

Moojase (2 µg) was incubated at room temperature for 40 min in the presence of different Chromogenix substrates at a final concentration of 0.4 mM: S-2238 (H-D-Phe-Pip-Arg-pNA·2HCl, substrate for thrombin), S-2366 (<Glu-Pro-Arg-pNA·HCl, substrate for factor XIa and activated protein C), S-2251 (H-D-Val-Leu-Lys-pNA·2HCl, substrate for plasmin and streptokinase-activated plasminogen), S-2302 (H-D-Pro-Phe-Arg-pNA·2HCl, substrate for plasma kallikrein, factor XIa, and factor XIIa), S-2222 (Bz-Ile-Glu(g-OR)-Gly-Arg-pNA·HCl R=H (50%) and R=CH_3_ (50%), substrate for factor Xa] and S-2765 (N-a-Z-D-Arg-Gly-Arg-pNA·2HCl, substrate for factor Xa). The reaction was monitored by absorbance at 405 nm using the SpectraMAX190 spectrophotometer (Molecular Devices). One unit of amidolytic activity (U) was defined as the increase of one absorbance unit at 405 nm, and the results were presented as specific activity (U/mg of Moojase).

### 5.14. Stability Studies

The stability of Moojase was initially assayed using its esterase activity on TAME. Briefly, the enzyme (5 µg) was preincubated for 30 min at different temperatures (4, 25, 37, 45, 65, and 100 °C) or pH buffers (3.0, 4.5, 6.0, 7.5, 9.0, and 10.5) at 37 °C, and then the esterase activity was assessed as described in Section 5.5. Results were then expressed as relative activity (%).

The thermostability of Moojase was also assessed by the thermofluor assay. This approach evaluates the effects of temperature-dependent protein unfolding by measuring the protein melting temperature (Tm) [47,62]. Moojase was screened for several solutions with different buffers, pH, and ionic strength formulations that promote protein solubility and stability. For that, we used two different kits: Solubility & Stability Screen, and Solubility & Stability Screen 2 (Hamptons Research, Aliso Viejo, CA, USA), following the manufacturer’s guidelines. The results were represented as a variation of Tm (∆Tm) according to the conditions tested. The values of ∆Tm (°C) were calculated according to Sartim et al. [47].

### 5.15. Statistical Analysis

The results were presented as mean ± SEM. Data were analyzed by one-way ANOVA followed by Tukey’s post-test, considering values of *p* < 0.05 as statistically significant. GraphPad Prism version 6.0 (GraphPad Software, La Jolla, CA, USA) was used for statistics.

## Figures and Tables

**Table 1:** Protein recovery and TAME activity for Moojase purification procedure.

Purification Step	Protein		Enzyme Activity	
	Total (mg)	Recovery (%)	TAME Activity (U/mg) *	Purification Factor
*B. moojeni* venom	200.0	100.0	1929.6	1
CM10 (CM Sepharose)	2.2	1.1	2120.0	1.09
Moojase (C18)	1.8	0.9	11,226.0	5.08

* Results expressed as specific activity, related to TAME units per milligram of protein (U/mg).

**Table 2:** Fibrinolytic activity.

Samples	Halo Diameter (mm) *
PBS (negative control)	0
Plasmin (20 µg) (positive control)	9.4 ± 1.2
Moojase (5 µg)	10.9 ± 1.2
Moojase (20 µg)	14.4 ± 1.4

* Results expressed as mean values ± SEM (*n* = 3).

**Table 3:** Platelet-aggregating activity.

Samples	Platelet Aggregation (%) *
ADP (20 µg) (positive control)	42.0 ± 8.0
Moojase (20 µg)	71.7 ± 1.1

* Results expressed as mean values ± SEM (*n* = 3).

## Supplementary Materials

The following are available online at http://www.mdpi.com/2072-6651/10/12/500/s1, Figure S1. Isoelectric focusing of Moojase. Moojase presented four protein bands, indicating different pI values ranging from 5.80 to 6.88. Figure S2. Molecular mass determination by Bruker Solarix 9.4T FTICR mass spectrometer. The mass spectra show the practical molecular mass calculated based on a 16-time charge. The box below presents the theoretical molecular mass of Moojase oxidized, as predicted by Sequence Editor software. Figure S3. Structural analysis of Moojase by mass spectrometry. (**A**) MALDI-TOF sequencing of Moojase, which covered 65.4% of the sequence template (Batroxobin). (**B**) Q-Exactive sequencing using Batroxobin as a template. The fragments that were sequenced in this methodology are marked in blue. (**C**–**E**) Sequencing of the fragments that presented the punctual mutations at positions 60, 110, and 124 of the template sequence.
