# Supplementary material for: New Insights on Moojase, a Thrombin-Like Serine Protease from Bothrops moojeni Snake Venom
Source: Toxins (Basel). 2018 Nov 28;10(12):500. doi: 10.3390/toxins10120500 (PMC6316876; doi:10.3390/toxins10120500)
Supplement: Supplementary file 1 [file toxins-10-00500-s001.pdf]

Supplementary Materials: New Insights on Moojase, a Thrombin-Like Serine Protease from *Bothrops moojeni* Snake Venom

Fernanda G. Amorim, Danilo L. Menaldo, Sante E. I. Carone, Thiago A. Silva, Marco A. Sartim, Edwin De Pauw, Loic Quinton and Suely V. Sampaio

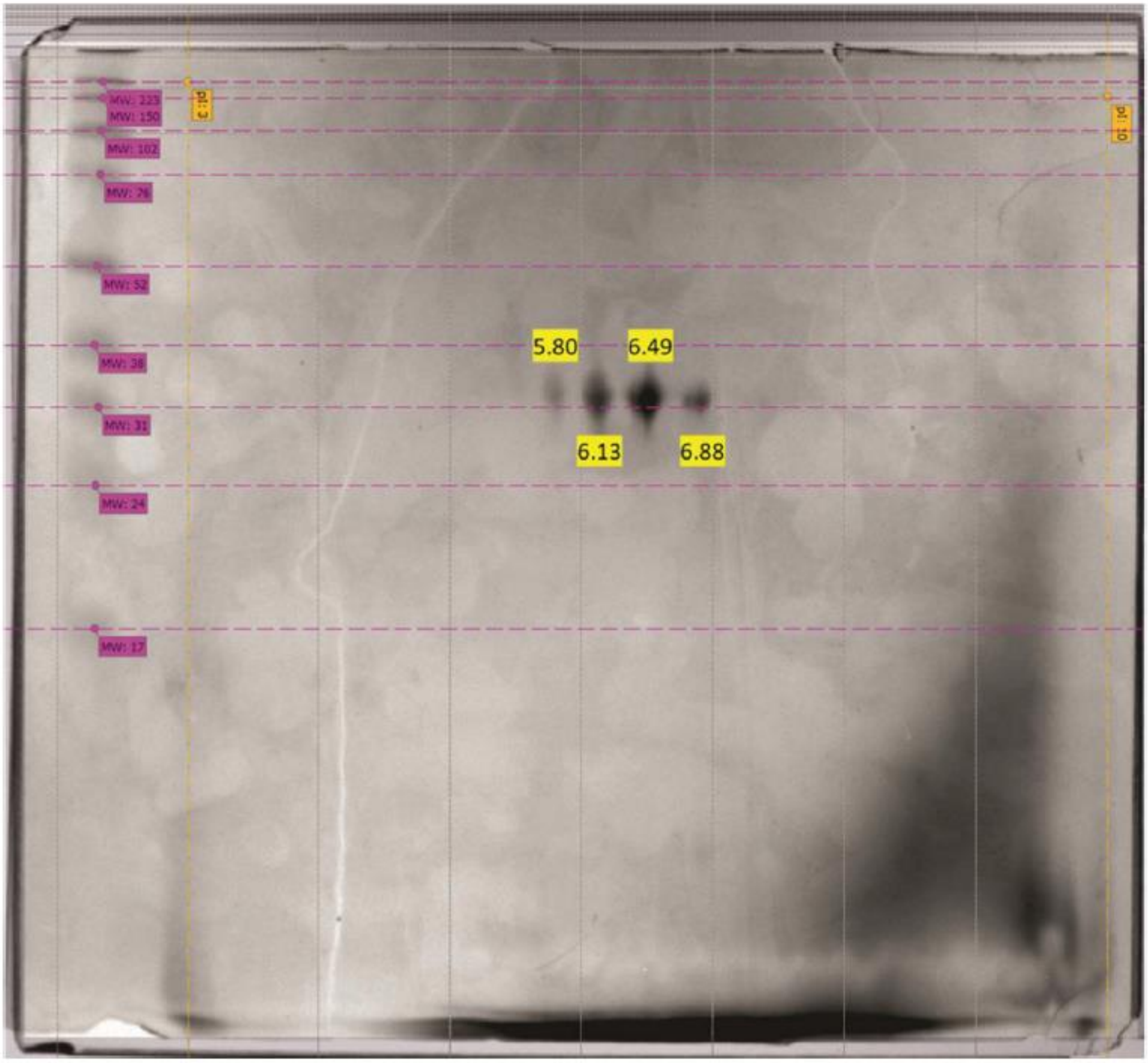

Figure S1. Isoelectric focusing of Moojase. Moojase presented four protein bands, indicating different pI values ranging from 5.80 to 6.88.

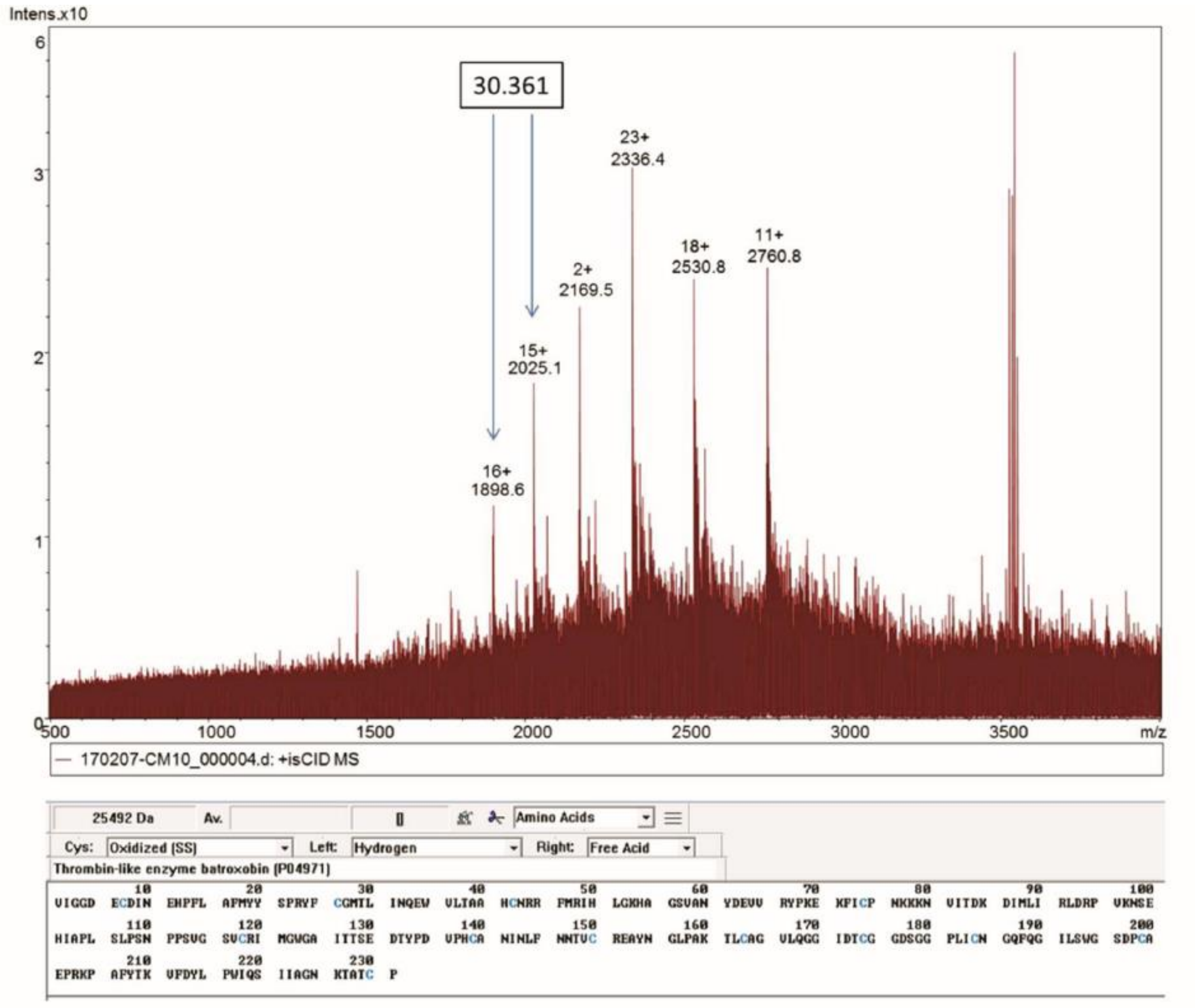

Figure S2. Molecular mass determination by Bruker Solarix 9.4T FTICR mass spectrometer. The mass spectra show the practical molecular mass calculated based on 16-time charge. The box below presents the theorical molecular mass of Moojase oxidized predicted by Sequence Editor software.

A

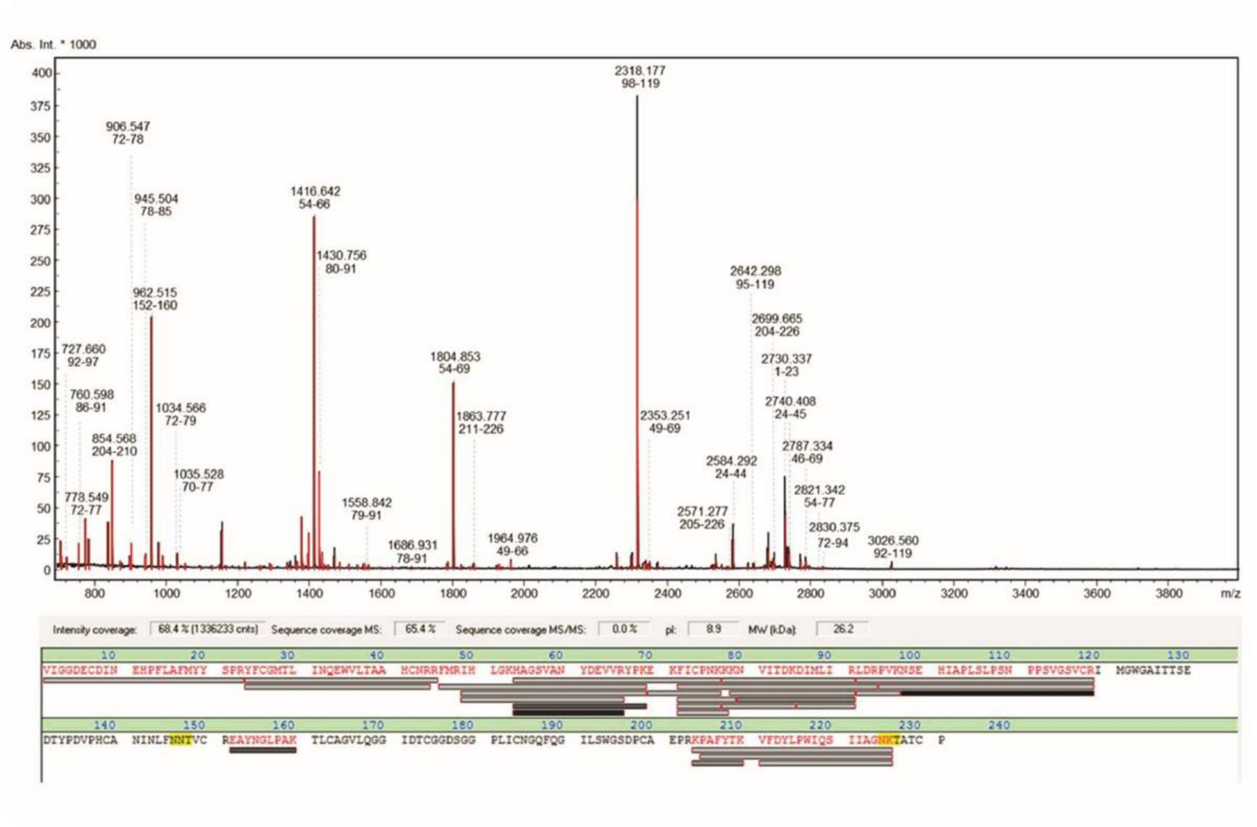
Abs. Int. * 1000
2318.177
98-119
906.547
72-78
945.504
78-85
1416.642
54-66
1430.756
80-91
962.515
152-160
727.660
92-97
760.598
86-91
854.568
204-210
1034.566
72-79
1035.528
70-77
778.549
72-77
1558.842
79-91
1686.931
78-91
1804.853
54-69
1863.777
211-226
1964.976
49-66
2353.251
49-69
2642.298
95-119
2699.665
204-226
2730.337
1-23
2740.408
24-45
2584.292
24-44
2571.277
205-226
2787.334
46-69
2821.342
54-77
2830.375
72-94
3026.560
92-119
m/z
Intensity coverage: 68.4 % (1336233 cnts)
Sequence coverage MS: 65.4 %
Sequence coverage MS/MS: 0.0 %
pI: 8.9
MW (kDa): 26.2

B

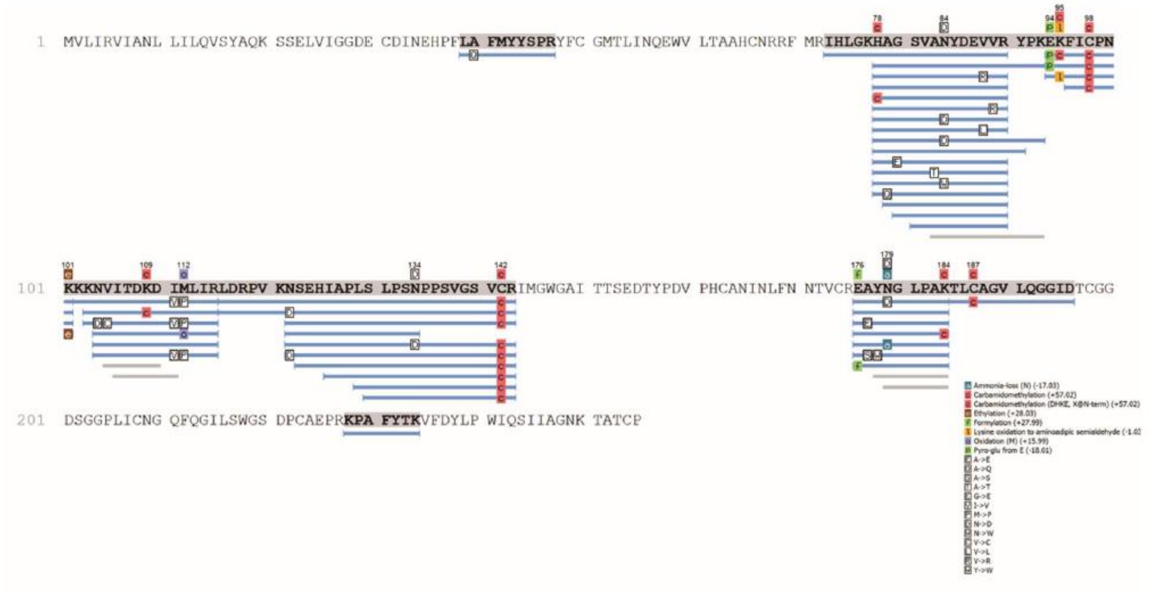

C

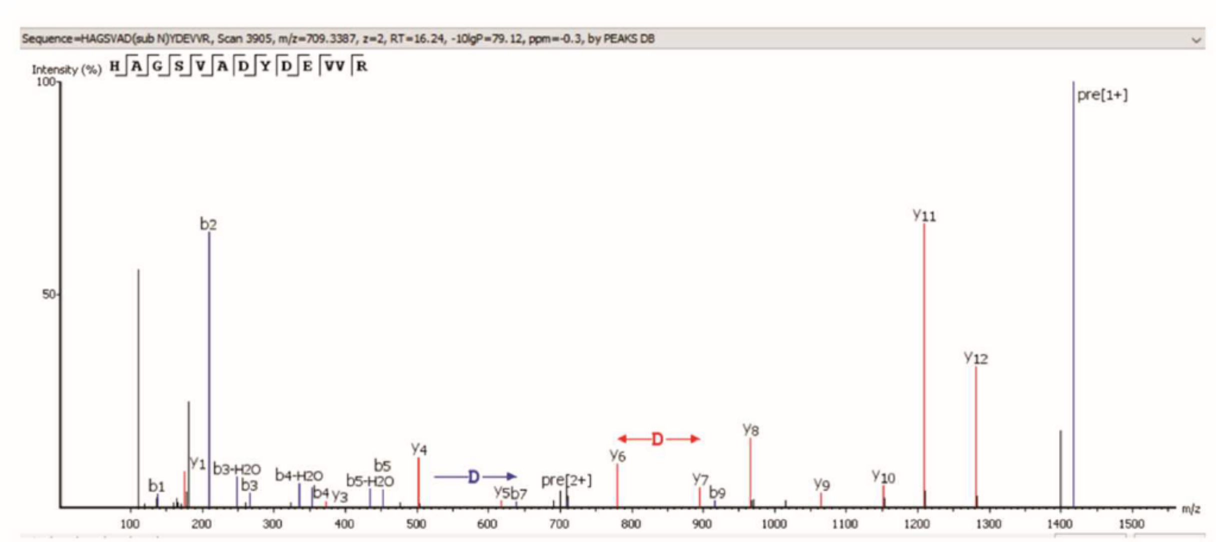

Sequence=HAGSVAD(sub N)YDEVVR, Scan 3905, m/z=709.3387, z=2, RT=16.24, -10lgP=79.12, ppm=-0.3, by PEAKS DB
Intensity (%)
H A G S V A D Y D E VV R
pre[1+]
y11
y12
b2
y8
y6
y4
y7
y9
y10
b1
y1
b3+H2O
b3
b4+H2O
b4
y3
b5
b5-H2O
y5b7
pre[2+]
b9
D
m/z

D

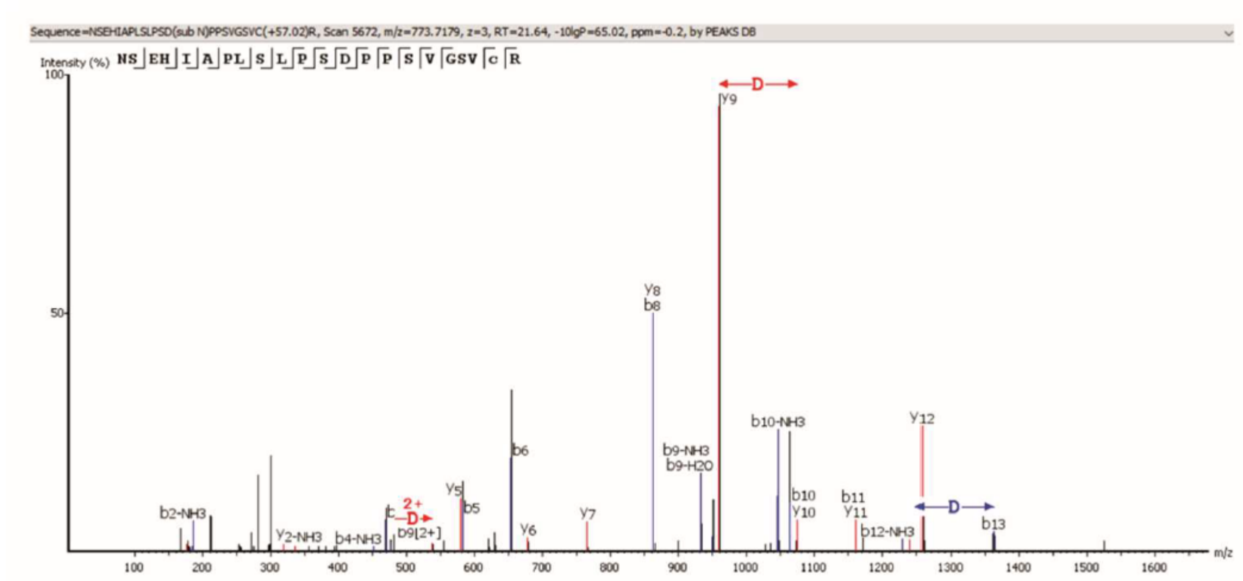

Sequence=NSEHIAPLSLPSD(sub N)PPSVGSVC(+57.02)R, Scan 5672, m/z=773.7179, z=3, RT=21.64, -10lgP=65.02, ppm=-0.2, by PEAKS DB
Intensity (%)
NS EH I A PL S L P S D P P S V GSV C R
y9
D
y8
b8
b10-NH3
y12
b6
b9-NH3
b9-H2O
b10
y10
b11
y11
b12-NH3
b13
y5
b5
y7
y6
b2-NH3
y2-NH3
b4-NH3
2+
b9[2+]
m/z

E

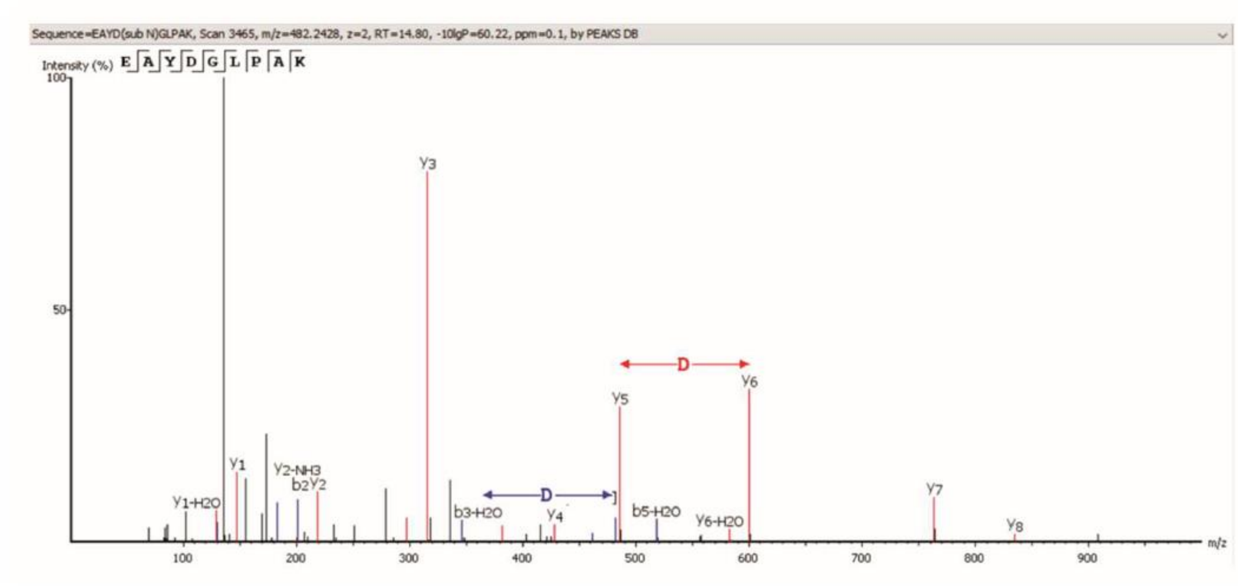

Figure S3. Structural analysis of Moojase by mass spectrometry. (**A**) MALDI-TOF sequencing of Moojase, which covered 65.4% of the sequence template (Batroxobin). (**B**) Q-Exactive sequencing using Batroxobin as a template. The fragments that were sequenced in this methodology are marked in blue. (**C**–**E**) Sequencing of the fragments that presented the punctual mutations at positions 60, 110 and 123 of the template sequence.
